# Biophysical Screens Identify Fragments That Bind to the Viral DNA-Binding Proteins EBNA1 and LANA

**DOI:** 10.3390/molecules25071760

**Published:** 2020-04-10

**Authors:** Troy E. Messick, Lois Tolvinski, Edward R. Zartler, Anna Moberg, Åsa Frostell, Garry R. Smith, Allen B. Reitz, Paul M. Lieberman

**Affiliations:** 1 The Wistar Institute, 3601 Spruce Street, Philadelphia, PA 19104, USA; ltolvinski@gmail.com; 2 Quantum Tessera Consulting, LLC, Collegeville, PA 19426, USA; teddyzartler@gmail.com; 3 GE Healthcare Bio-Sciences AB, Björkgatan 30, SE-751 84 Uppsala, Sweden; Anna.Moberg@ge.com (A.M.); asafrostell@gmail.com (Å.F.); 4 Fox Chase Chemical Diversity Center, Inc., 3805 Old Easton Road, Doylestown, PA 18902, USA; gssynthesis@gmail.com (G.R.S.); AReitz@fc-cdci.com (A.B.R.)

**Keywords:** Epstein–Barr virus, Epstein–Barr nuclear antigen 1, Kaposi’s sarcoma associated herpesvirus (KSHV), latency-associated nuclear antigen, protein–DNA interaction, saturation transfer difference-nuclear magnetic resonance, surface plasmon resonance, fragment-based lead discovery

## Abstract

The human gamma-herpesviruses Epstein–Barr virus (EBV) (HHV-4) and Kaposi’s sarcoma-associated herpesvirus (KSHV) (HHV-8) are responsible for a number of diseases, including various types of cancer. Epstein–Barr nuclear antigen 1 (EBNA1) from EBV and latency-associated nuclear antigen (LANA) from KSHV are viral-encoded DNA-binding proteins that are essential for the replication and maintenance of their respective viral genomes during latent, oncogenic infection. As such, EBNA1 and LANA are attractive targets for the development of small-molecule inhibitors. To this end, we performed a biophysical screen of EBNA1 and LANA using a fragment library by saturation transfer difference (STD)–NMR spectroscopy and surface plasmon resonance (SPR). We identified and validated a number of unique fragment hits that bind to EBNA1 or LANA. We also determined the high-resolution crystal structure of one fragment bound to EBNA1. Results from this screening cascade provide new chemical starting points for the further development of potent inhibitors for this class of viral proteins.

## 1. Introduction

Small-molecule disruption of macromolecular interactions is becoming an increasingly important strategy for developing new therapies to treat human diseases. However, discovery of these inhibitors can be particularly challenging due to the diversity of interactions that must be satisfied by a small molecule to compete with the interactions between macromolecules [1]. Another challenge is the relatively limited collection of existing high-throughput libraries compared with theoretical chemical space. Despite these challenges, there have been a number of successes—including clinically—of inhibitors of protein–protein interactions (PPIs) [2,3,4].

Another type of macromolecular interaction that can be targeted by small-molecule inhibitors and deserves more focus is protein–nucleic acid. Protein–nucleic acid interactions are important in numerous human diseases including cancer and infectious disease. For example, transcription factors account for approximately 20% of all identified oncogenes. However, efforts to discover small-molecule inhibitors that specifically target these protein–DNA interactions remain in a relatively early stage [5]. Many viruses control viral gene expression and DNA replication using site-specific DNA-binding proteins [6], which can be targeted by small molecules [7].

Epstein–Barr virus (EBV) and Kaposi’s sarcoma-associated herpesvirus (KSHV) are collectively responsible for almost 2% of all human cancers [8,9,10,11]. EBV (HHV-4) is etiologically associated with a number of lymphoid malignancies including Burkitt’s lymphoma, Non-Hodgkin lymphoma, Hodgkin lymphoma, and the solid neoplasms nasopharyngeal carcinoma and subtypes of gastric carcinoma [12,13]. KSHV (HHV-8) is responsible for all forms of Kaposi’s sarcoma, pleural effusion lymphoma and multicentric Castleman’s disease [14].

EBV and KSHV establish lifelong persistence in host lymphocytes as circular DNA molecules, referred to as episomes. Critical for maintaining viral episomes are the homologous Epstein–Barr nuclear antigen 1 (EBNA1) and KSHV latency-associated nuclear antigen (LANA). EBNA1 and LANA bind to specific genetic elements on the viral episome, where they recruit cellular replication machinery to initiate DNA replication, regulate viral gene expression, and tether viral genomes to host chromosomes during mitotic segregation [6]. The DNA-binding domains of EBNA1 and LANA share a similar overall backbone architecture with other viral proteins like human herpesvirus 6A (HHV-6A) immediate early protein 2 (IE2) and human papillomavirus (HPV) E2, but no other human proteins [6,15,16,17]. Thus, their critical roles in viral maintenance and function in promoting continued proliferation of host cells and the lack of human orthologs make EBNA1 and LANA attractive targets for the development of small-molecule inhibitors to treat viral-associated disease, especially cancer.

The strategy of fragment-based drug discovery (FBDD) uses biophysical methods to detect the binding of fragments with a molecular weight of ≤300 as starting points to generate drug leads [18,19]. In FBDD, small fragment libraries of less than 1000 fragments can often be screened efficiently [20]. Although fragment hits usually have weak binding affinities (µM–mM) for the target, structural data can be used to optimize inhibitors by increasing potency of compounds from fragments that already have favorable physicochemical properties [21,22].

Previously, we reported the discovery of an EBNA1 inhibitor series using FBDD from a pilot fragment screen of 100 fragments using X-ray crystallography [7]. The 100-fragment library was chosen based on in silico docking to a site adjacent to the site identified by X-ray crystallography [23]. To provide additional chemical starting points using a more unbiased initial screen for EBNA1 and to identify initial fragment hits for LANA, we report here the screening of a 1000 fragment library by saturation transfer difference (STD)–NMR and surface plasmon resonance (SPR). We also report the crystal structure of one fragment bound to EBNA1. The results from this study may provide further chemical starting points for the development of potent inhibitors of EBNA1 or LANA, members of an important class of DNA-binding proteins.

## 2. Results

**Figure 1:**
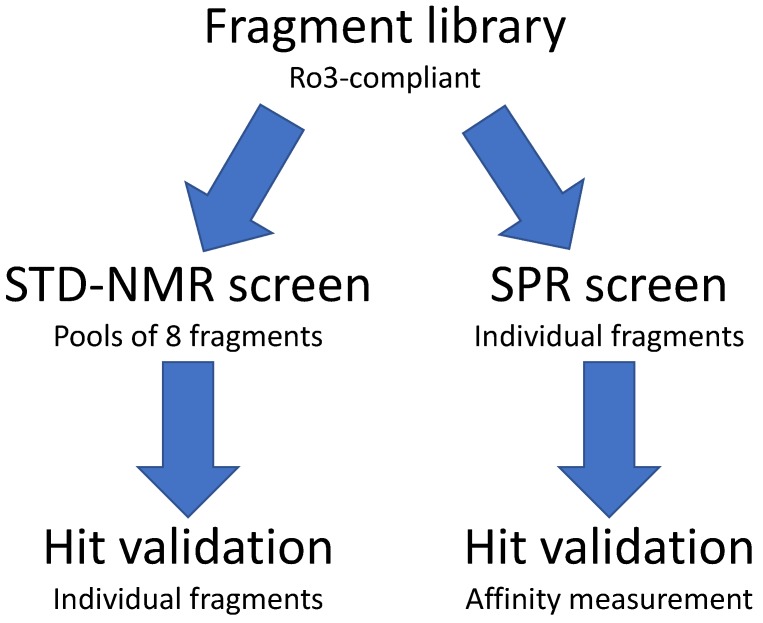
Fragment screening strategy to identify fragments that bind Epstein–Barr nuclear antigen 1 (EBNA1) and KSHV latency-associated nuclear antigen (LANA). STD–NMR, saturation transfer difference–NMR; SPR, surface plasmon resonance.

We implemented a biophysical screening strategy to identify chemical starting points for the development of inhibitors of EBNA1 and LANA (Figure 1). The “Rule of 3”-compliant Maybridge fragment library of 1000 fragments was chosen primarily because unwanted reactive functionalities were absent, and each member was experimentally tested to have >1 mM aqueous solubility. This later requirement is thought to decrease the false-positive hit rate from aggregated compounds and provide starting points with favorable physicochemical properties. We screened the entire library against EBNA1 and LANA using both STD–NMR spectroscopy and SPR. 

### 2.1. STD–NMR Screen

One thousand fragments were grouped into 125 pools of 8 fragments based on predicted non-overlapping NMR chemical shifts. NMR parameters were optimized to keep the acquisition time to less than 1 hour with good signal to noise. All pools were successfully run. 

During the EBNA1 screen, we used fragment VK0044/CC34301 as a control. This fragment was identified from a previous X-ray crystal screen [7]. VK0044 was used to optimize and validate the method and demonstrate protein stability and signal quality over 5 days. During the screen, VK0044 was run every 25 pools as a control to monitor reproducibility of the assay. The ^1^H–NMR spectra from the compound mixtures were compared with the predicted spectra from the individual fragments, which allowed the deconvolution of the experimental data in an automated fashion.

**Figure 2:**
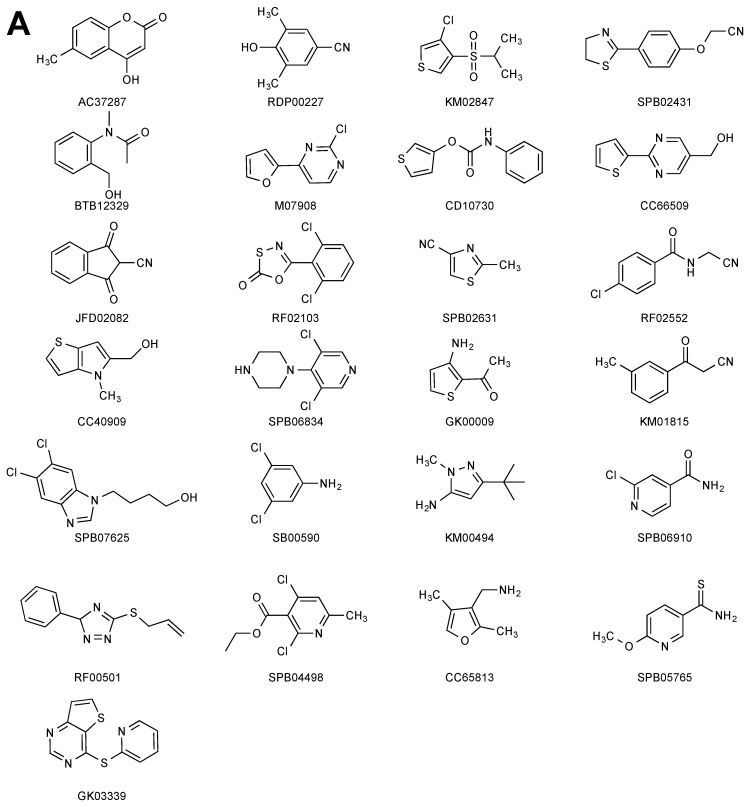

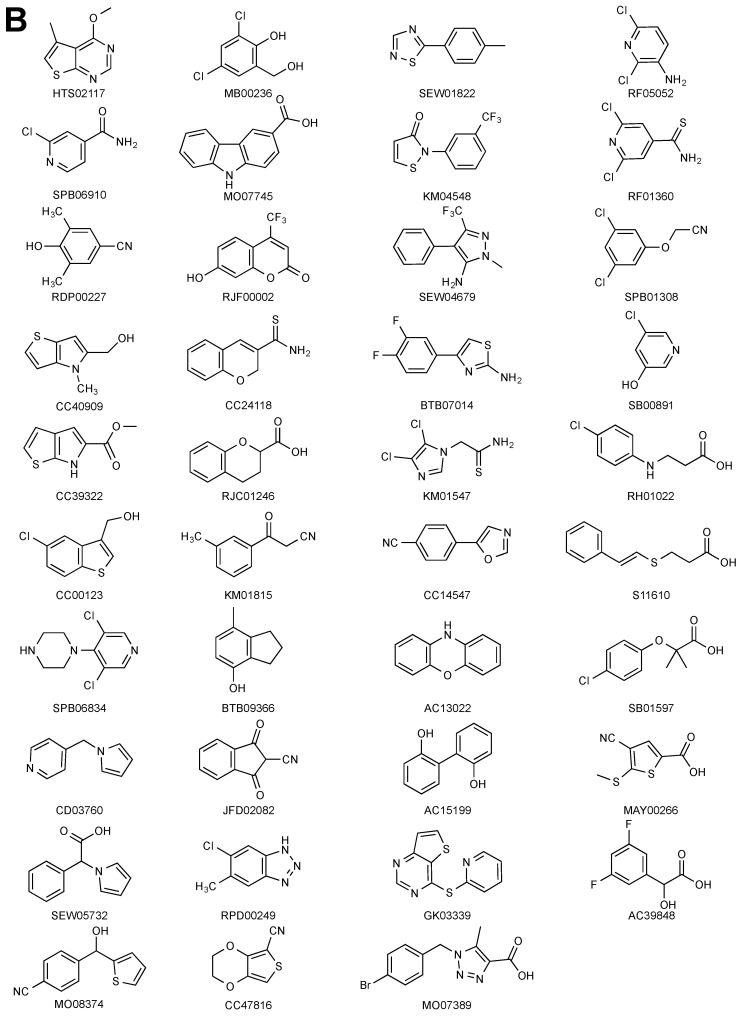
Validated fragment hits from the NMR-STD screen. (**A**) Structures of the validated EBNA1 fragment hits. (**B**) Structures of the validated LANA fragment hits.

Fifty-eight pools were found to have putative hits. These were divided into two groups based on manual inspection of peak intensities: Thirty-six potential hits in 27 pools were assigned to high confidence group (>75% matched peaks) and 46 potential hits in the other 31 pools were assigned to a low confidence group (<75% matched peaks). Fifty-one potential hits (36 high confidence and 15 low confidence) were selected for further hit validation. The ^1^H spectra for each of the individual hits were collected (reference) and compared with the STD spectra (difference) under the same conditions as the original screen (Figure S1). Twenty-five fragments were confirmed as hits, although four fragments were classified as binding very weakly, i.e., not all peaks from the reference spectrum were present in the difference spectrum (Figure 2A). Twenty-six fragments were not confirmed as binders. Overall, this screen yielded a 2.5% hit rate.

For the LANA screen, 44 pools had putative hits. Using a similar process as before, 57 fragments were selected for further hit validation. When tested individually, 39 fragments were confirmed as binders (Figure 2B, Figure S2), while 18 were not. This screen yielded a hit rate of 3.9%.

### 2.2. SPR Screen

SPR experiments were performed on a Biacore™ S200 instrument (GE Healthcare). We found that primary amine coupling of the wild-type EBNA1 DNA-binding domain (DBD) via lysine residues had significantly reduced binding to DNA. The structure of EBNA1 and LANA bound to DNA suggest that lysine residues are important for DNA binding. As an alternative, we developed a thiol coupling method to immobilize EBNA1 and LANA to the Biacore sensor chip. We synthesized a construct that expressed a version of EBNA1 and LANA DNA-binding domains with the internal cysteines mutated to serine and Cys-Ala-Cys added to the C terminus (EBNA1-CAC and LANA-CAC). These versions of EBNA1 and LANA after immobilization to the CM5 Biacore sensor chip via thiol coupling were capable of binding DNA with affinities comparable to solution-based studies with the DBD of each protein.

**Figure 3:**
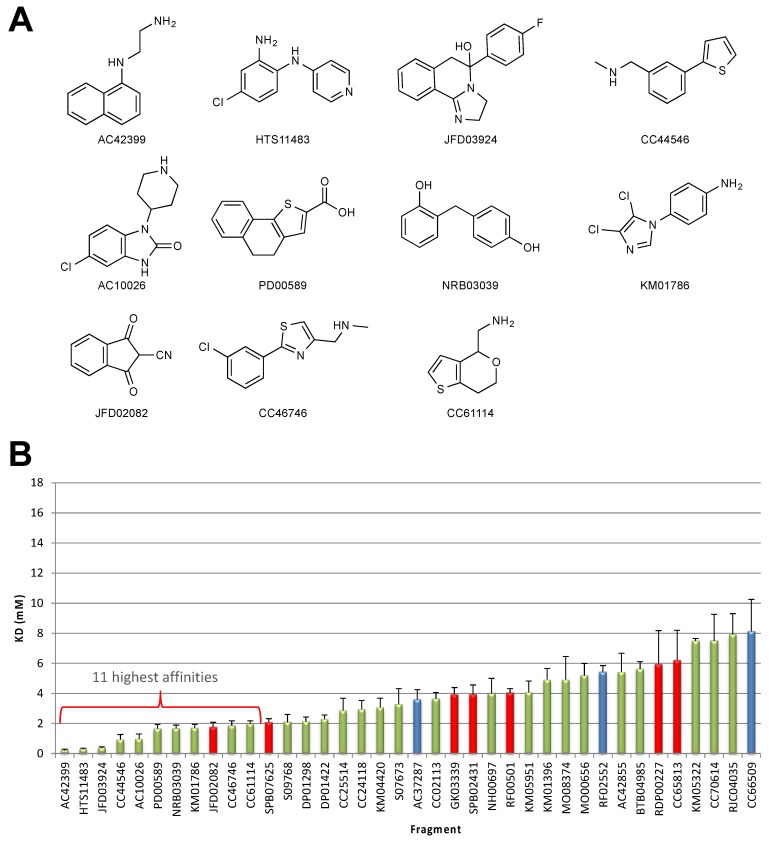

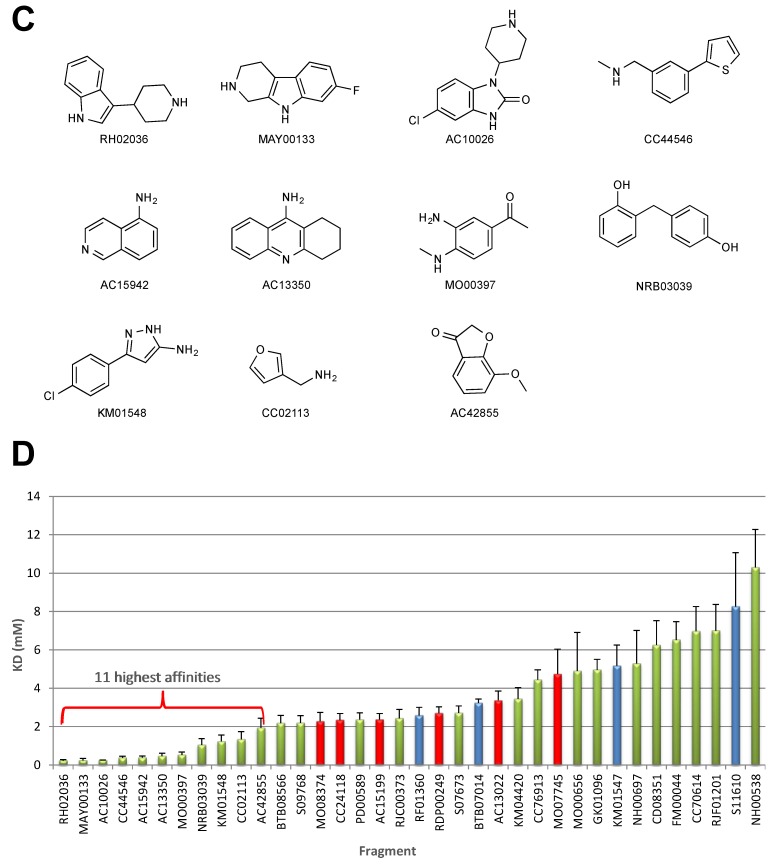
Validated fragment hits from the SPR screen. (**A**) Structures of the top 11 fragments that bind with the highest affinity to EBNA1. (**B**) Bar graph showing the binding affinities of the top EBNA1 fragments. Green bars are SPR-only fragment hits. Red bars are overlapping hits from the STD–NMR screen. Blue bars are initial hits from the STD–NMR screen that were not subsequently validated. Error bars (fitting error) show assay robustness, typically approximately, or below, 25% variation. (**C**) Structures of the top 11 fragments that bind with the highest affinity to LANA. (**D**) Bar graph showing the binding affinities of the top EBNA1 fragments. Colors and error bars are as described in (**B**).

The 1000 fragments from the library were screened at a concentration of 1 mM (5% DMSO). Hits were selected on the basis of the following criteria: a relative binding response normalized with respect to the positive control, no indication that multiple fragments were bound, R > R_max_ (R = Response units, R_max_ = the maximum binding capacity based on control fragment) and sensorgram shapes consistent with fragment binding (rapid on and off rates). Based on these criteria, 94 EBNA1 fragments and 104 LANA fragments were selected for further validation to measure binding affinities, which were determined to range from 0.2 to >10 mM. The structures of the 11 highest binding fragments for EBNA1 and LANA and the binding affinities are shown in Figure 3.

### 2.3. X-Ray Crystallography

**Figure 4:**
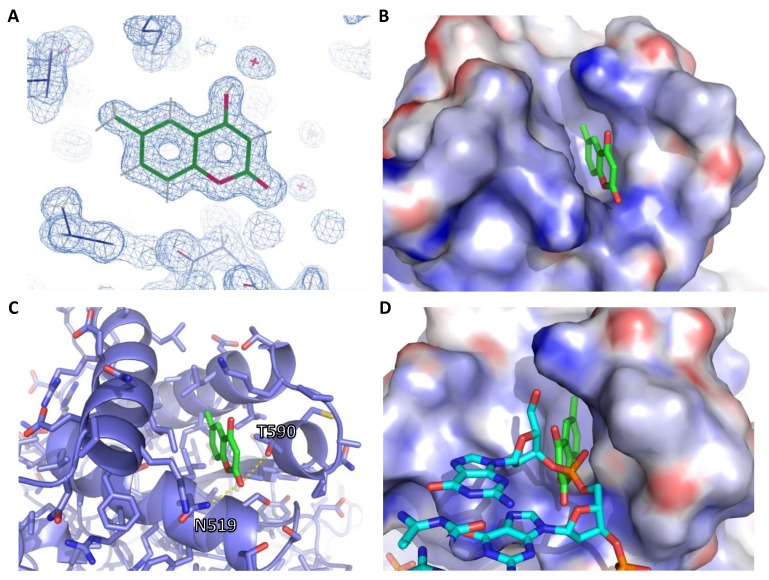
High-resolution crystal structure of EBNA1 bound to AC37287. (**A**) 2F_o_–F_c_ electron density map of AC37287 (green). The map was contoured at 1.0 σ. (**B**) Electrostatic surface representation of AC37287 bound to EBNA1. (**C**) Cartoon and stick representation showing hydrogen bonding interaction between D519 and AC37287. (**D**) Superposition of DNA structure on the crystal structure of AC37287 bound to EBNA1.

We determined the high-resolution crystal structure of one fragment bound to EBNA1 (Figure 4). We soaked the fragment AC37287 into preformed crystals of EBNA1 (468–607). The crystal of AC37287 diffracted to 1.3 Ångstroms and was solved by isomorphous replacement. The electron density around AC38287 is unambiguous at this resolution (Figure 4A). The aliphatic portion of AC37287 binds into a deep hydrophobic pocket made up of the hydrophobic residues I481, L520, L582, V483 and I593 (Figure 4B). The lactone is sandwiched between residues N519 and T590 within the range of a hydrogen bond of N519 (Figure 4C). This molecule binds in a region that is important for DNA binding (Figure 4D). Superposition of the crystal structures of both EBNA1 bound to AC37287 and to DNA shows that AC37287 binds to a region that sterically clashes with the phosphate backbone of DNA.

## 3. Discussion

We previously performed a fragment screen of EBNA1 with a small library of 100 fragments using X-ray crystallography [7]. The fragment library was selected based on in silico docking to a site thought to be important for DNA binding. However, the fragment screen revealed a previously unknown “hot spot” site (site 2) to which 12 fragments were bound. By merging two fragments, we identified a compound series that inhibited DNA binding in vitro and in several cell-based assays. However, because of the high density of basic residues in the site selected for docking, we noted that the small 100 fragment library was enriched with carboxylic acid-containing fragments. To carry out a less biased fragment screen, we screened a larger collection of 1000 fragments to identify additional potential binding sites or provide additional chemical starting points.

Our screening strategy accomplishes two objectives. First, applying orthogonal biophysical methods is thought to increase the confidence in fragments that bind using both methods, as well as not miss any potential hits that may bind in only one assay. Second, we wanted to develop inhibitors against both targets and take advantage of any structural similarity and fragment binding modes that may develop during the course of the hit-to-lead campaigns. 

**Figure 5:**
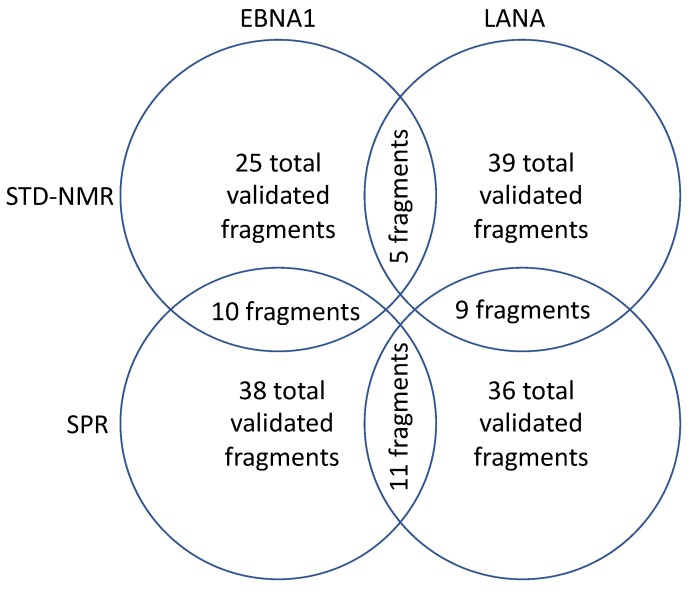
An analysis of the fragments from the screen.

An analysis of the fragments that bind in both assays shows that there is some overlap, bolstering the validity of the methods (Figure 5). Ten fragments were in common between STD–NMR and SPR methods in the EBNA1 program and nine fragments were in common between both methods for the LANA program. However, many fragments only bound in one assay, including AC37287, for which we obtained the crystal structure. We believe this result argues for “casting a wider net” by utilizing both methods. These results are consistent with the observations of others that different biophysical methods often yield divergent fragment hits [24,25].

Another interesting result was the analysis of fragments that bound using the same method. For example, in the STD–NMR method, five fragments were in common between the EBNA1 and LANA. Further, in the SPR method, 11 fragments were in common between EBNA1 and LANA. One benefit of the FBDD approach is the low fragment complexity that allows fragments to bind to a few key molecular features on a protein. Even though EBNA1 and LANA share a similar fold, the sequence similarity is less than 35% (sequence identity <25%). These scaffolds may hit a hot spot, which enables them bind to a few directed attractive interactions with the protein so that biophysical detection of target binding is possible.

Due to the diverse nature of the starting library, any detectable structure–activity relationship (SAR) in the validated hits may be weak. However, there are a few common functional groups that were observed. In the STD–NMR screen of EBNA1, six fragments contain exocyclic nitriles. In addition, we observed four fragments that were dichloro-aromatics and two fragments that were mono-aromatics. In the STD–NMR screen of LANA, seven fragments contained carboxylic acids, six fragments contained dichloro-aromatic groups, and five fragments contained mono-aromatic groups.

Schulz, Empting and colleagues reported a fragment screen with 720 fragments against LANA using SPR and differential scanning fluorimetry (DSF) [26]. They made derivatives of an initial hit, an imidazoloaniline fragment, that showed dose-dependent activity in a biochemical fluorescence polarization (FP) LANA DNA disruption assay. They also demonstrated preliminary SAR in the series and modeled where a pyridinyl-triazolo-benzoic acid compound may be binding.

We attempted to obtain co-crystal structures of other hits from the screen with EBNA1 but were unsuccessful. This may be due to the EBNA1 crystal form and the fragments that are able to access differing areas of the protein. For example, surfaces that are important for crystal formation may occlude fragment binding. To obtain information about where these fragments may be binding, we could collect heteronuclear single-quantum coherence (HSQC) NMR spectra with ^15^N-labeled protein.

The structures of the DNA-binding domains of EBNA1 and LANA reveal opportunities to inhibit the interaction with DNA with small molecules [16,17]. The stable dimeric core of antiparallel β sheets is surrounded by three critical α-helices that mediate important contacts with the DNA. In particular, EBNA1 Lys477, Asn519, Lys586 and Thr590 and LANA Lys1030, Lys1070, and Ala1129 at the base of the α-helices form a pocket that sandwiches the DNA phosphate backbone and make sequence-specific contacts with guanine residues. We identified a coumarin molecule, AC37287, that binds in this pocket of EBNA1 with the potential to inhibit DNA binding. This pocket is made up of a core of hydrophobic residues—Ile481, Leu485, Leu520 and Leu582—surrounded by the polar residues Asn519 and Thr590, which make hydrogen bonding contacts with a DNA phosphate (Figure 4C,D). This is the first time a bicyclic heteroaromatic group or a non-acidic-containing fragment has been observed in a crystal structure in this pocket. The fragments identified here may bind to ideal protein motifs and may serve as desirable starting points for further optimization. For example, linking a coumarin molecule to the acetylene of the previously identified series, i.e., VK-1248 or VK-1760 [7], may lead to a new chemical series that disrupts EBNA1–DNA interactions.

## 4. Materials and Methods

### 4.1. Protein Expression and Purification

For the STD–NMR screen, EBNA1 (aa 459–607) and LANA (972–1110) proteins were expressed in *Escherichia coli* as His-tag-SUMO fusion proteins and purified using a method similar to that described previously [15,27]. Briefly, protein was expressed using the autoinduction method for ~24 h at 25 °C [28]. Bacteria cultures were centrifuged and resuspended in a high-salt solution (25 mM Tris, 1 M NaCl, 10% glycerol, 5 mM 2-mercaptoethanol). Cells were lysed with lysozyme and sonication, in the presence of PMSF and 0.1% Tween 20. After centrifugation to remove insoluble cell debris, the cell lysate was applied to a Nickel column, washed with >20 column volumes of high-salt buffer and eluted with a buffer containing 300 mM imidazole, 1 M NaCl, 10% glycerol and 5 mM 2-mercaptoethanol. Fractions containing fusion protein were confirmed by SDS-PAGE, concentrated and applied to a Superdex prep-grade 75 size-exclusion column, equilibrated with a high-salt buffer to remove the imidazole. Fractions containing fusion protein were pooled and incubated with SUMO protease overnight at 4 °C. The cleaved protein was applied to the nickel column to remove the His-SUMO and any uncut fusion protein. The flow through was collected and concentrated. 

In the last step to purify protein for the STD–NMR screen, the size-exclusion column was equilibrated with a solution containing 10 mM Tris-d_11_, 1M NaCl, 0.5 mM DTT-d_11_, pD 7.9 in D_2_O. EBNA1 and LANA were concentrated to 178 and 377 μM, respectively. For the SPR screen, the same procedure was used to purify versions of EBNA1-CAC and LANA-CAC. For the last step, the size-exclusion column was equilibrated with a solution containing 10 mM Tris, 1 M NaCl, and 0.5 mM DTT, pH 7.9.

### 4.2. Pooling for NMR Screen

One thousand fragments were grouped into 125 pools of 8 fragments each. Pooling was performed based on predicted non-overlapping chemical shifts using the NMR prediction algorithms implemented in the Perch software (Perch Solutions). Briefly, a Monte Carlo algorithm was implemented to minimize NMR signal overlap [29]. The threshold for pairwise peak overlap, i.e., minimum distance between non-overlapping peaks, was set at 0.1 ppm.

### 4.3. STD–NMR Screen

All NMR experiments were performed at 25 °C in 3 mm NMR tubes, using a Bruker Avance III 500 MHz spectrometer (Bruker Corporation, Billerica, MA, USA) equipped with a TCI CryoProbe, and Bruker TopSpin 3.1 software for data processing. Each fragment was resuspended in DMSO-d_6_, combined in pools of 8 fragments and diluted to a final concentration per fragment of 500 μM diluted with a binding solution containing 10 mM Tris-d_11_, 200 mM NaCl, 0.5 mM DTT-d_11_, pD 7.9 D_2_O. The protein was diluted with binding solution to a concentration of 4 μM based on the signal and the stability of protein and the control compound VK0044/CC34301 over 5 days. The NMR parameters were optimized to keep the acquisition time to less than 1 hour with good signal to noise as follows: 3 s saturation at 0.2 ppm, 0.3 s recovery delay and 512 scans. For the EBNA1 project, VK0044 was used as a positive control. A volume of 500 μM was chosen for the ligand concentration to keep the DMSO <5%. 

Data were collected successfully from all 125 pools. During the EBNA1 screen, VK0044 was run every 25 pools as a control in order to ensure highly reproducible results. The ^1^H–NMR spectra from the compound mixtures were compared with the predicted spectra from the individual fragments, which allowed the deconvolution of the experimental data in a semi-automated fashion using the Mnova software, version 10.0.2 (Mestrelab Research). After the initial screen, potential hit fragments were validated by collecting data from the individual fragments incubated with protein under the same conditions as the original screen.

### 4.4. SPR Screen

EBNA1-CAC and LANA-CAC were immobilized to the CM5 Biacore sensor chip (GE Healthcare, Marlborough, MA, USA) via thiol coupling according to the manufacturer’s instructions (GE Healthcare) and subsequently confirmed to be capable of binding DNA. Assay conditions were optimized so that EBNA-CAC and LANA-CAC could be run simultaneously in flow cells 2 and 4. Flow cells 1 and 3 were used as reference cells (blank immobilization). Approximately 1800 RU of protein was immobilized in a buffer containing 20 mM HEPES, 200 mM NaCl, and 1 mM MgCl_2_, pH 7.1. For the screen, we used running buffer containing 50 mM Tris, 200 mM NaCl, 1 mM MgCl_2_, 0.05% Surfactant P20, and 5% DMSO, pH 7.1. The flow rate was 30 μl/min and the contact time was 20 s. To regenerate the flow cell, 4 M NaCl for 30 s was used.

To validate fragments from the primary screen, an affinity assay was performed. Fragment concentrations that were assayed were 79, 119, 178, 267, 400, 600, and 900 μM. Binding sensorgrams were processed by first subtracting the binding response recorded from the control surface (reference flow cell), followed by solvent correction. To determine the binding affinity (K_D_ values), data sets were fitted to a simple 1:1 interaction model by fitting the response 7 s after injection during the association phase to a single-site binding isotherm. Affinities were assessed using a global R_max_ determined by reference compounds. Sensorgrams of the top 11 fragments from the EBNA1 screen are displayed in Figure S3. Sensorgrams of the top 11 fragments from the LANA screen are shown in Figure S4. Example dose-response curves from the SPR experiments using SPB07625 with EBNA1 and S09768 with LANA are shown in Figure S5 and Figure S6, respectively. 

### 4.5. X-Ray Crystallography

Crystals were grown using the vapor diffusion method in 24–48 h using purified EBNA1 (468–607) mixed in a 2 μl:2 μl ratio with reservoir solution (50 mM MES, pH 6.5, 0–150 mM NaCl, and 10 mM DTT). Crystals were transferred to a solution containing 25–30% glycerol and 0.2 μl of 50 mM of AC37287, incubated for 4 h and flash frozen for data collection. Data were collected on the 21BM beamline at the Argonne Light Source (ALS) at Argonne National Laboratory. Data were indexed, reduced, and scaled using HKL3000. The structures were solved by isomorphous replacement. Models were refined in PHENIX, version 1.17, using simulated annealing, minimization, and individual B-factor refinement. Between refinement cycles, the model was manually rebuilt using the program Coot. Data collection and refinement statistics are summarized in Table 1.

## Figures and Tables

**Table 1:** Data collection and refinement statistics of the EBNA1:AC37287 cocrystal structure.

	EBNA1:AC37287 PDB Code 6VH6
Spacegroup	P2_1_2_1_2_1_
Wavelength	0.97919
a, b, c (Å) α, β, γ (°)	59.52, 68.34, 69.69 90.00, 90.00, 90.00
Resolution (Å)	50.0-1.30 (1.32-1.30)
I/σ	26.4 (1.03)
Completeness	99.1% (91.9%)
Redundancy	6.4 (3.1)
Reflections	69927 (3190)
R_work_/R_free_	0.1500/0.1608
Wilson B factor (Å^2^)	14.6
Bond lengths (Å)	0.009
Bond angles (°)	1.004
Ramachandran favored	99%
Ramachandran allowed	1%
Ramachandran outliers	0%
Clash score	4.0
Average B, all atoms (Å^2^)	23.0
Number of protein atoms	4572
Number of solvent atoms	454
Number of ligand atoms	21
Total number of atoms	5047

## Supplementary Materials

The following are avaiable online. Figure S1: Validated hits from the EBNA1 STD-NMR screen. Figure S2: Validated hits from the LANA STD-NMR screen. Figure S3: Sensorgrams and relative dissociation constants (KD) of top 11 compounds from the EBNA1 screen. Figure S4: Sensorgrams and relative dissociation constants (KD) of top 11 compounds from the LANA screen. Figure S5: Example dose-response curve from the SPB07625 performed with EBNA1. Figure S6: Example dose-response curve from the S09768 performed with LANA.

## Abbreviations

DBD	DNA-binding domain
DSF	Differential Scanning Fluorimetry
EBV	Epstein-Barr Virus
EBNA1	Epstein–Barr Nuclear Antigen 1
FBDD	Fragment-Based Drug Discovery
FP	Fluorescence Polarization
IE2	Immediate Early Protein 2
HHV-4	Human Herpesvirus-4
HHV-6A	Human Herpesvirus-6A
HHV-8	Human Herpesvirus-8
HPV	Human Papillomavirus
HSQC	Heteronuclear Single-Quantum Coherence
KSHV	Kaposi’s Sarcoma-associated Herpesvirus
LANA	Latency-Associated Nuclear Antigen
PPI	Protein–Protein Interaction
Ro3	Rule of Three
SAR	Structure Activity Relationship
SPR	Surface Plasmon Resonance
STD–NMR	Saturation Transfer Difference-Nuclear Magnetic Resonance
