# Supplementary material for: Biophysical Screens Identify Fragments That Bind to the Viral DNA-Binding Proteins EBNA1 and LANA
Source: Molecules. 2020 Apr 10;25(7):1760. doi: 10.3390/molecules25071760 (PMC7180839; doi:10.3390/molecules25071760)
Supplement: Supplementary file 1 [file molecules-25-01760-s001.pdf]

Supplement for:

Biophysical Screens Identify Fragments that Bind to the Viral DNA Binding Proteins EBNA1 and LANA

Troy E. Messick [1,*], Lois Tolvinski [1], Edward R. Zartler [2], Anna Moberg [3], Åsa Frostell [3], Garry R. Smith [4], Allen B. Reitz [4] and Paul M. Lieberman [1,*]

[1] The Wistar Institute, 3601 Spruce Street, Philadelphia, PA 19104, USA; ltolvinski@gmail.com (L.T.)
[2] Quantum Tessera Consulting, LLC, Collegeville, PA 19426, USA; teddyzartler@gmail.com (E.R.Z.)
[3] GE Healthcare Bio-Sciences AB, Björkgatan 30, SE-751 84 Uppsala, Sweden; Anna.Moberg@ge.com (A.M.), asafrostell@gmail.com (A.F.)
[4] Fox Chase Chemical Diversity Center, Inc., 3805 Old Easton Road, Doylestown, PA, 18902, USA; gssynthesis@gmail.com (G.R.S.), AReitz@fc-cdci.com (A.B.R.)
*Correspondence: Lieberman@wistar.org (P.M.L.), tmessick@wistar.org (T.E.M.);
Tel.: 215-898-9523 (P.M.L.), 215-898-3896 (T.E.M.)

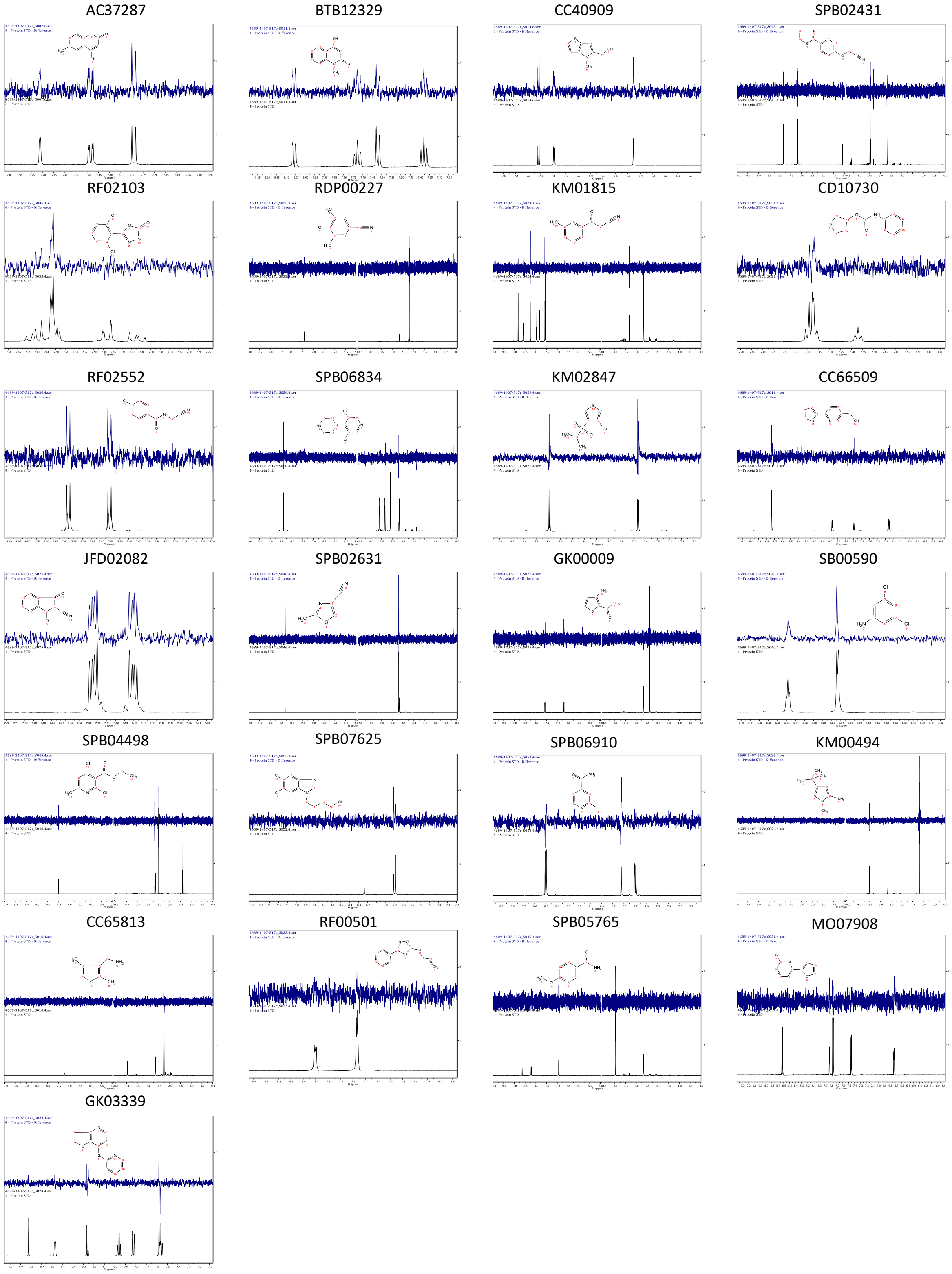

Figure S1 Validated hits from the EBNA1 STD–NMR screen. The reference spectrum is shown in black, the difference spectrum in blue.

Fragment screening of EBNA1 and LANA

AC13022

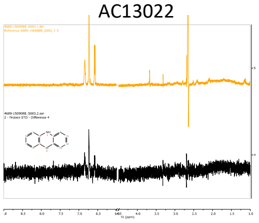

AC15942

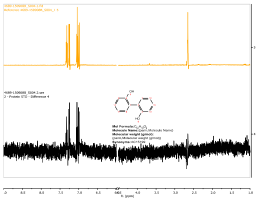

AC38948

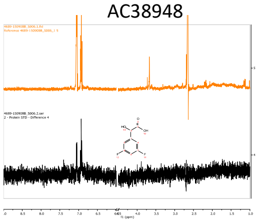

BTB07014

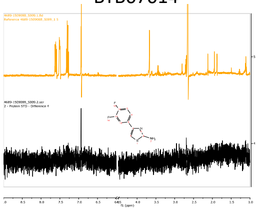

BTB09366

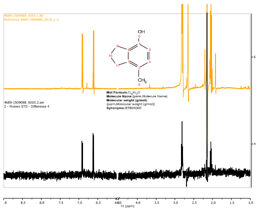

CC00123

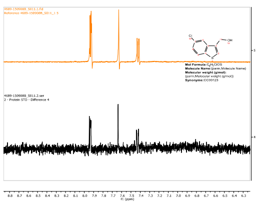

CC14547

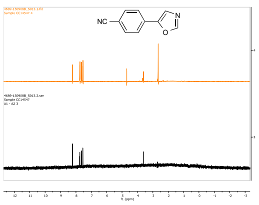

CC24118

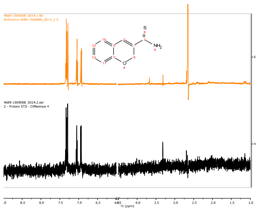

CC39322

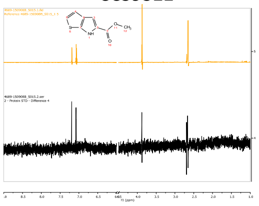

CC40909

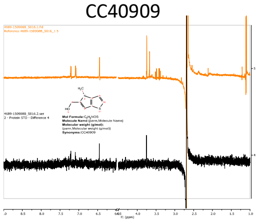

CC47816

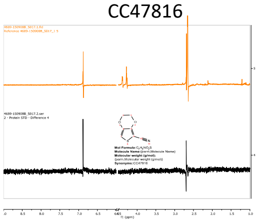

CD03760

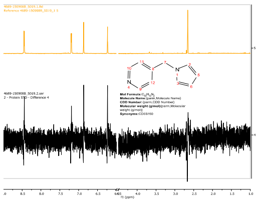

GK03339

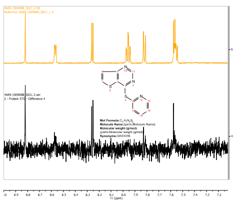

HTS02117

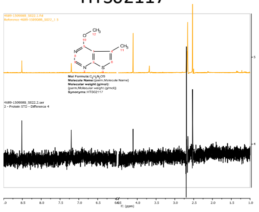

KM01547

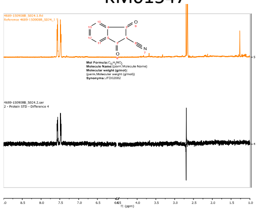

JFD02082

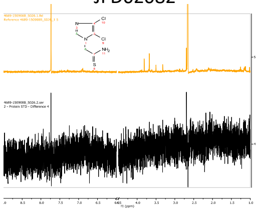

KM01815

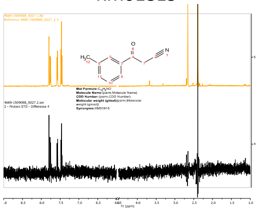

KM04548

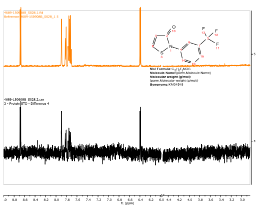

MAY00266

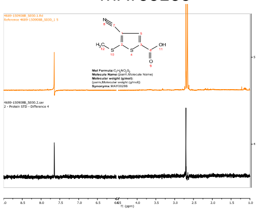

MB00236

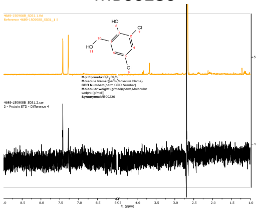

MO07389

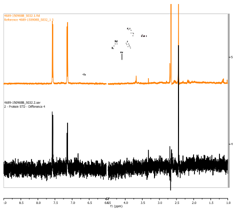

MO07745

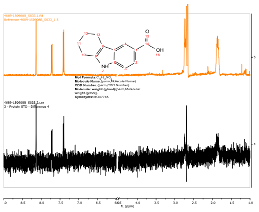

MO08374

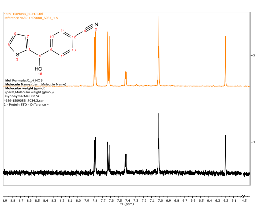

RDP00227

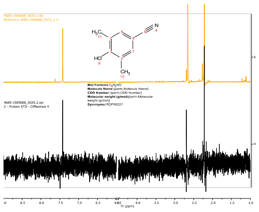

RDP00249

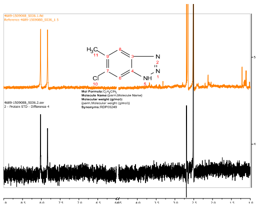

RF01360

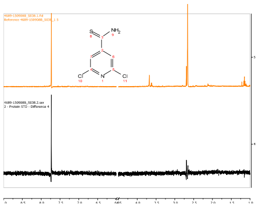

RF05052

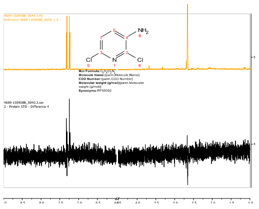

RH01022

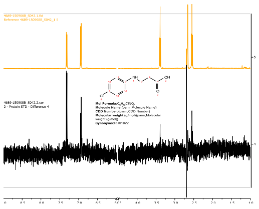

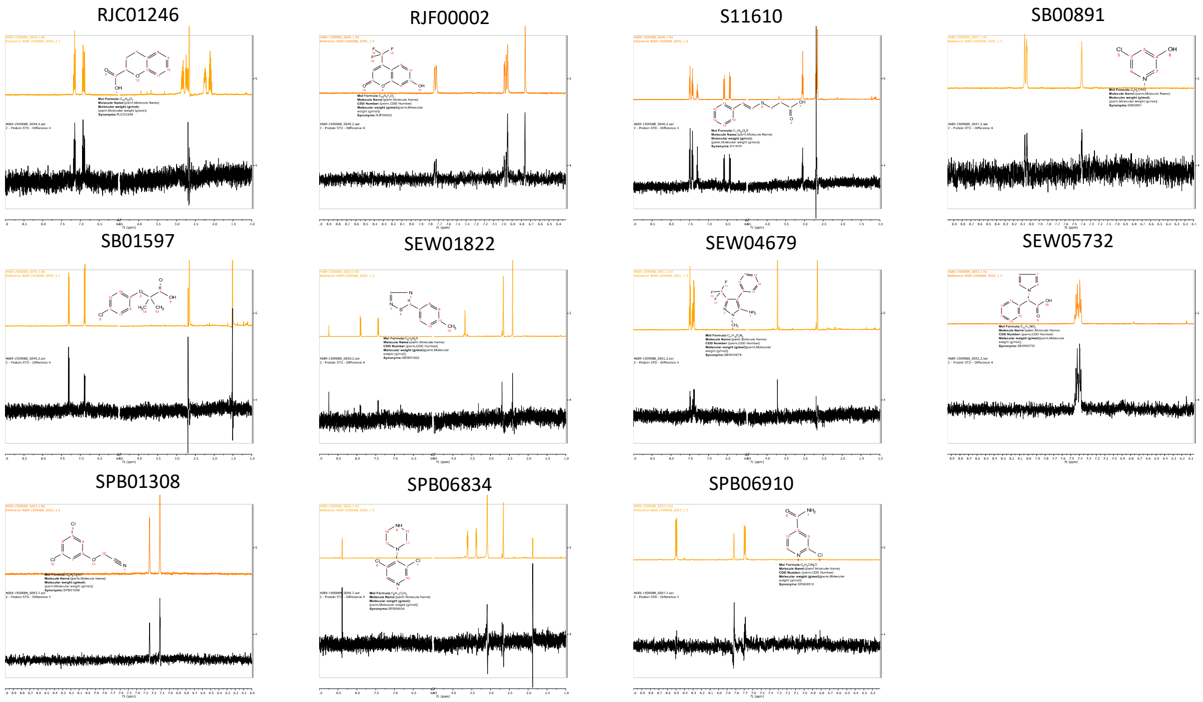

Figure S2. Validated hits from the LANA STD–NMR screen. The reference spectrum is shown in yellow, the difference spectrum in black.

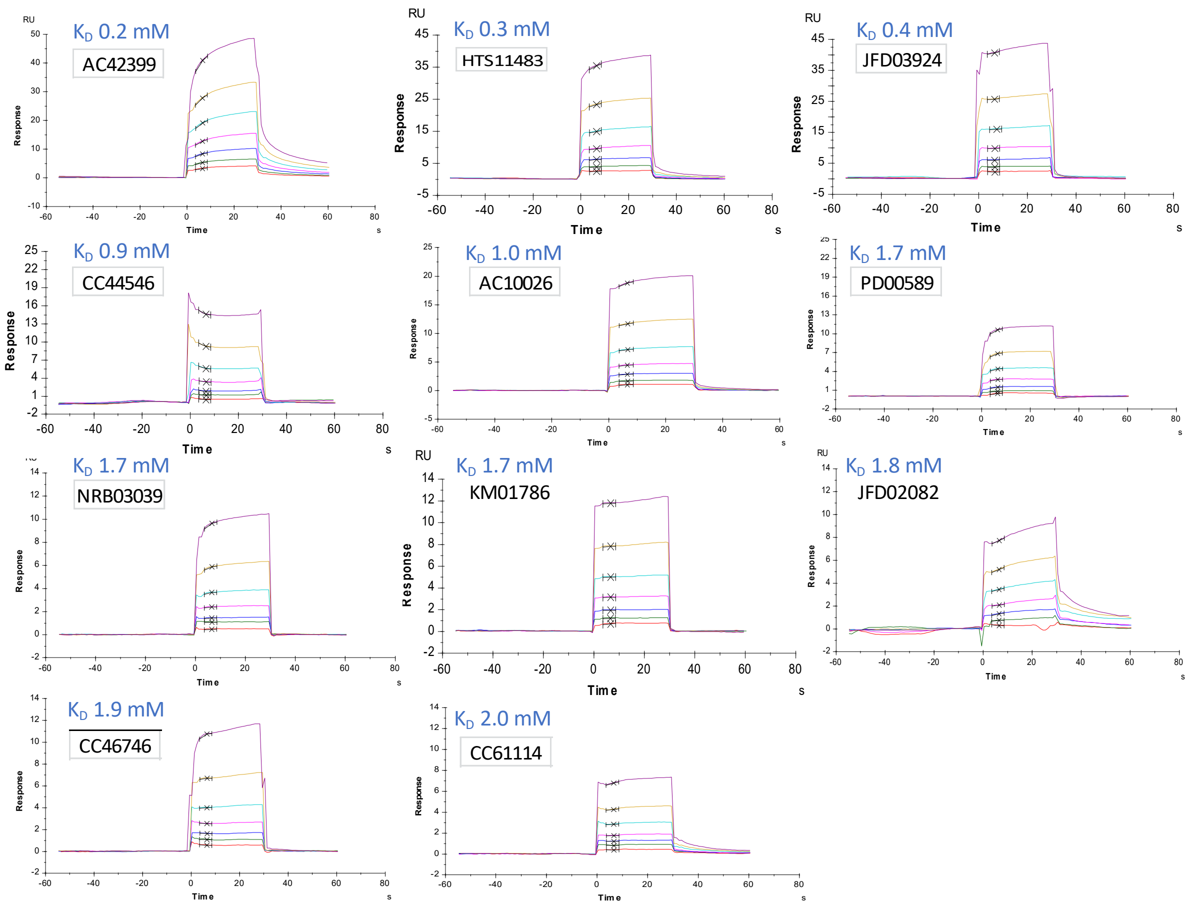

Figure S3. Sensorgrams and relative dissociation constants (K_d) of top 11 compounds from the EBNA1 screen.

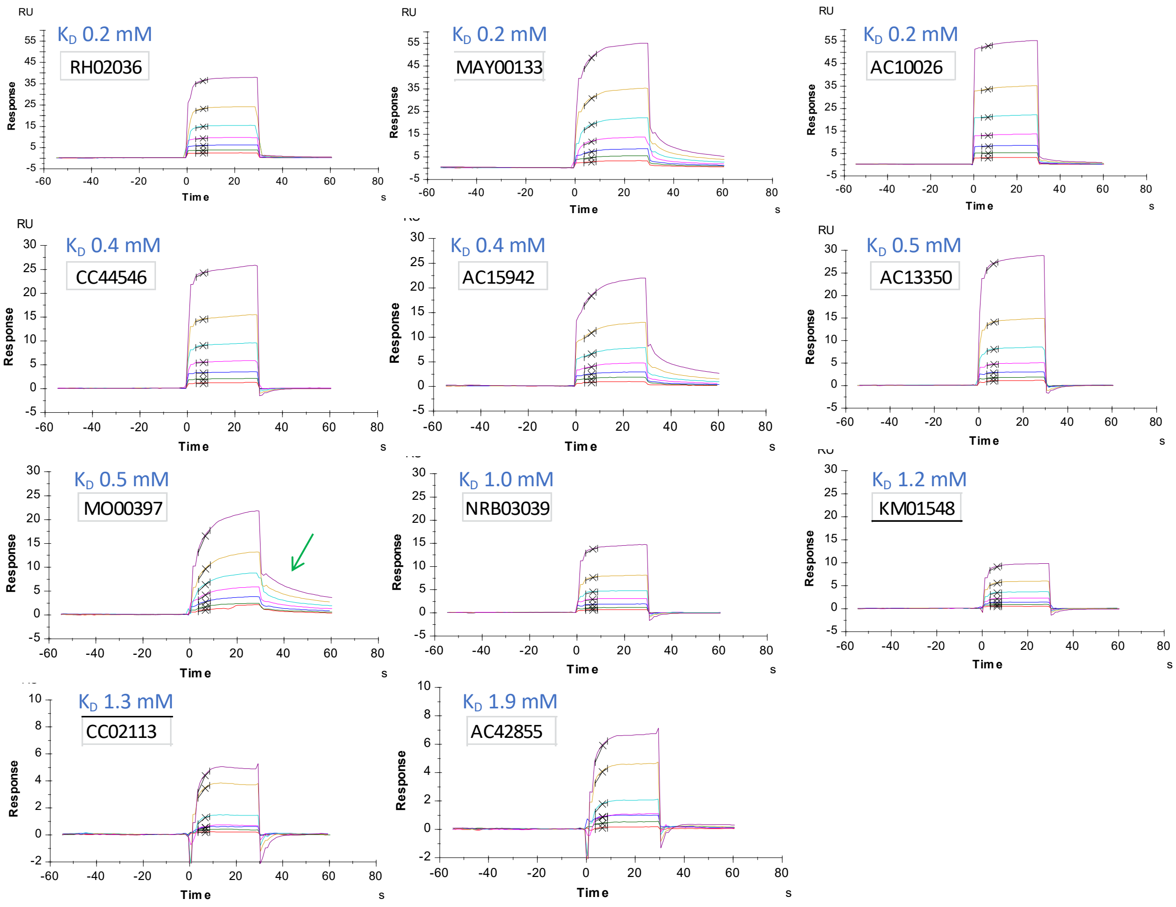

Figure S4. Sensorgrams and relative dissociation constants (K_d) of top 11 compounds from the LANA screen.

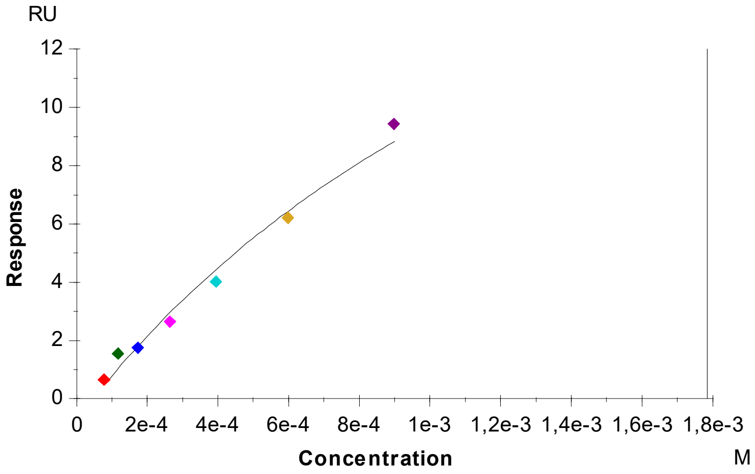

Figure S5. Example dose-response curve from the SPB07625 performed with EBNA1. The calculated K_D of SPB07625 was 2.1 mM.

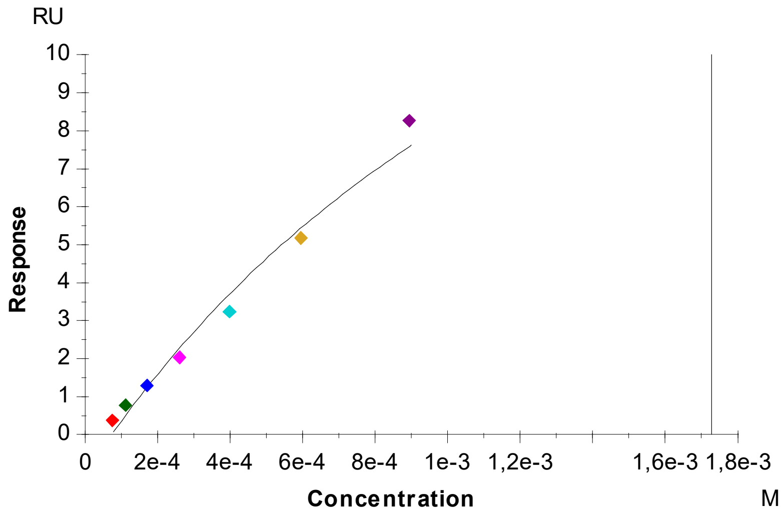

Figure S6. Example dose-response curve from the S09768 performed with LANA. The calculated K_D of S09768 was 2.2 mM.
